# Bacterial gastroenteritis in the world of culture-independent diagnostic testing: a study to evaluate the kinetics of bacterial shedding by culture and CIDT

**DOI:** 10.1128/spectrum.00227-25

**Published:** 2025-05-22

**Authors:** Nathan K. McCartney, Surangi H. Thilakarathna, Tara Hluchy, Dylan R. Pillai, Linda Chui, Byron M. Berenger

**Affiliations:** 1 Department of Pathology and Laboratory Medicine, Cumming School of Medicine, University of Calgary 574842, Calgary, Alberta, Canada; 2 Department of Laboratory Medicine and Pathology, University of Alberta 536883, Edmonton, Alberta, Canada; 3 Environmental Public Health, Alberta Health Services 3146, Edmonton, Alberta, Canada; 4 Microbiology, Diagnostic and Scientific Centre, Alberta Precision Laboratories 590576 , Calgary, Alberta, Canada; 5 Alberta Public Health Laboratory (ProvLab), Alberta Precision Laboratories 590576 , Calgary, Alberta, Canada; Central Texas Veterans Health Care System, Temple, Texas, USA

**Keywords:** gastroenteritis, polymerase chain reaction, bacteriology, *Campylobacter*, *Salmonella*, Shiga toxin-producing *Escherichia coli*, feces, culture

## Abstract

Reliable microbiological diagnosis is important for patient and public health management of bacterial gastroenteritis. Clinical laboratories are adopting culture-independent diagnostic tests (CIDT) widely. We examined the kinetics of bacterial shedding by CIDT and culture following an episode of acute gastroenteritis with one of three prevalent pathogens. The primary outcome was the proportion of samples that tested CIDT-positive or culture-positive at defined time intervals after clinical diagnosis. This longitudinal observational study was conducted in Calgary Health Zone (Alberta, Canada) between 2019 and 2022. Eligible adult patients (≥18 years) were consented and enrolled after a routine public health investigation. Stool samples were submitted at weeks 2, 3, 4, 5, and 6 post-diagnosis. Stools were tested using the commercial nucleic acid-based BD MAX Enteric Bacterial Panel (CIDT method) and routine culture methods. Thirty-seven of 83 enrolled individuals (45%) submitted at least one additional stool. Diagnostic specimens were positive for *Campylobacter* (*n* = 19), *Salmonella* (*n* = 8), and Shiga toxin-producing *Escherichia coli* (STEC, *n* = 10). Median duration of CIDT positivity was 42, 29, and 22 days for *Campylobacter, Salmonella,* and STEC, respectively. Median duration of culture positivity was 29, 29, and 19.5 days, respectively. Differences between CIDT and culture positivity over time were not statistically significant for *Campylobacter* (*P* = 0.23), *Salmonella* (*P* = 0.89), and STEC (*P* = 0.20). A negative CIDT result was 100% predictive of a culture-negative result. Comparing CIDT and culture, no differences in shedding were found between methods in this pilot study. Further larger studies would help better understand the kinetics of CIDT positivity and clinical implications.

**IMPORTANCE:**

This study contributes to the limited literature comparing the shedding of common bacterial enteropathogens by culture-independent diagnostic testing (CIDT) methods in comparison to conventional culture. Many clinical microbiology laboratories have implemented CIDT methods for screening stool specimens due to the slower turnaround times and variable sensitivity of culture. Public health agencies often require a negative culture for return-to-work clearance in special situations or sensitive occupations, including food handlers. In our study, we demonstrated that there were no significant differences in the duration of shedding between test methods for three common bacterial pathogens, *Campylobacter*, *Salmonella,* and Shiga toxin-producing *Escherichia coli*. Furthermore, a negative CIDT result was 100% predictive of a negative culture result. This study contributes to the growing body of literature documenting the clinical and public health utility of CIDT in bacterial gastroenteritis.

## INTRODUCTION

Bacterial gastroenteritis has a significant health, social, and economic impact worldwide. An estimated 19.5 million cases of gastroenteritis occur each year in Canada alone (1), with only 4 million cases reported to public health authorities annually, with the most commonly identified bacterial enteropathogens being *Salmonella*, *Campylobacter,* and Shiga toxin-producing *Escherichia coli* (STEC) (2).

Stool culture remains the gold standard for microbiological diagnosis of bacterial gastroenteritis, yet due to slow and labor-intensive protocols with variable performance, many clinical laboratories are employing culture-independent diagnostic testing (CIDT) (3, 4). Several commercial multiplex PCR kits for common enteropathogens are available, including the BD MAX Enteric Bacterial Panel. Comparative performance data with other available commercial assays have previously been demonstrated, and with good performance in a clinical setting (5, 6). These syndromic panels can be performed directly on unpreserved stool, which provides a diagnostic result within several hours, allowing for rapid identification of cases and possible outbreak scenarios. While cases may be more rapidly identified, several limitations to CIDT remain. Culture of isolates is essential to allow for molecular typing to aid in outbreak detection and management. Without isolation of the causative bacteria, antimicrobial susceptibility testing cannot be performed for both surveillance and clinical management. Conventional and molecular typing methods used for public health epidemiology would also be limited, which have also been identified as concerns by numerous public health agencies (7). Per current recommendations, stool specimens that screen positive by CIDT should be cultured to support important public health surveillance efforts (8, 9).

Clearance of culturable organisms from stool is an important tool for public health agencies in case management, including for the return to activity in sensitive occupations or situations, such as food handlers, and for children attending daycare. There is limited data comparing the performance and duration of positivity by CIDT and traditional culture-based methods. CIDT positivity is expected to persist beyond viable culture due to the shedding of microbial DNA, which does not necessarily correlate to live, infectious organisms (10–12).

To our knowledge, this is the first study to evaluate shedding of three prevalent enteric pathogens in stool by CIDT and culture. The goal of our study was to better understand the kinetics of nucleic acid detection compared with conventional culture for cases of bacterial gastroenteritis. In this longitudinal observational study, we evaluated serial stool submissions from individuals with a positive stool culture for *Campylobacter*, *Salmonella,* or STEC for up to 6 weeks after submission of a diagnostic specimen. We assessed the duration of test positivity by two methods, culture and CIDT (BD MAX Enteric Bacterial Panel). In addition, specimens were tested by organism-specific confirmatory laboratory-developed quantitative PCR (qPCR).

## MATERIALS AND METHODS

### Study design and participants

**Fig 1:**
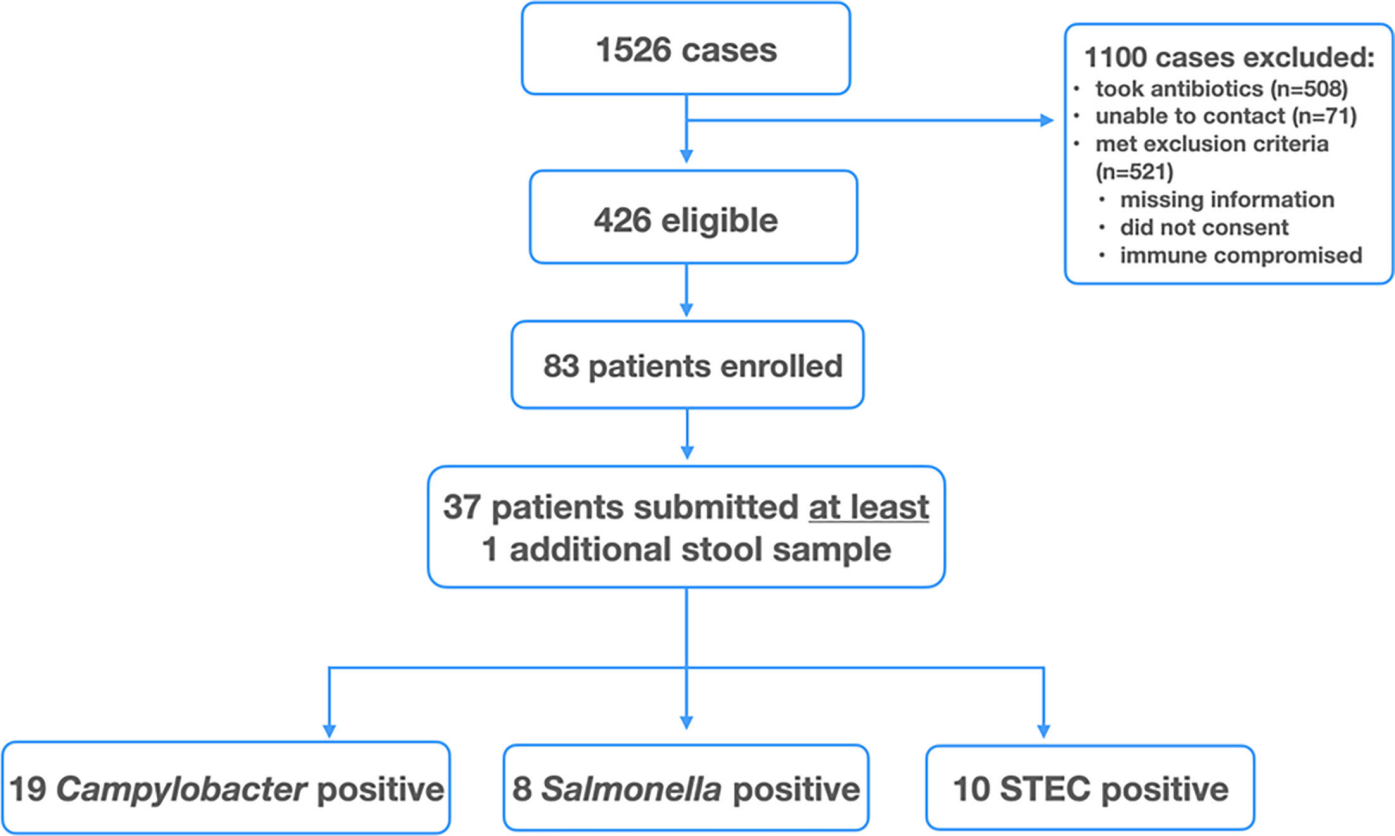
Flow diagram showing selection of study participants.

In this prospective observational study, adult (≥18 years of age at time of diagnostic stool submission) community patients in Calgary, Alberta, Canada with a first positive stool culture for *Campylobacter*, *Salmonella,* or STEC tested at Alberta Precision Laboratories for clinical diagnosis between 2019 and 2022 were eligible for enrollment. Later in the study, individuals residing in Edmonton, Alberta, tested at DynaLIFE Medical Laboratories in Edmonton were also included. During routine public health follow-up, including clinical, exposure, and occupational history, individuals were asked by the Public Health Inspector if they would be interested in participating. Individuals who were interested were subsequently contacted and enrolled by the study team unless they had received antibiotics since symptom onset or met other exclusion criteria (Fig. 1). Patients who were excluded from sensitive situations or occupations by Public Health were not included in the study. Follow-up questionnaires were conducted to monitor for symptom resolution. Participants were asked to submit weekly stool samples at week 2, 3, 4, 5, and 6 after symptom onset and to answer questions on the study lab requisition about whether they still had symptoms or took any medications. Participants who provided at least one stool sample after the diagnostic sample were included in the analysis. There was variability in the numbers and timing of stool specimens received. To ensure an adequate number of specimens, we accepted any stools submitted by participants, with the maximum being 61 days post-onset.

#### Specimen collection

Subsequent stool samples were submitted by participants using a sterile container without transport media from a kit provided to each participant with stool collection instructions (see Supplemental material). Samples were either dropped off at a laboratory collection site or picked up by a study-funded courier and transported to the testing laboratory. Stool specimens were refrigerated as per BD MAX manufacturer’s instructions for up to 5 days prior to processing.

### Testing methods

Initial unpreserved diagnostic stools were tested with the BD MAX Enteric Bacterial Panel as per the manufacturer’s specifications. All stools that were positive by BD MAX were reflexed to culture for the specific enteropathogen (see below). All study stools were tested by both BD MAX and organism-specific culture based on the initial diagnostic result.

*Campylobacter*-positive specimens were planted to *Campylobacter* blood-free agar (Dalynn Biologicals, Calgary, AB) and incubated in microaerophilic conditions at 42°C for 48–72 hours. Colonies were identified using colony morphology, Gram stain, oxidase, and MALDI-TOF (VitekMS, Biomérieux, Marcy-l’Étoile, France). *Salmonella-*positive stools were planted to CHROMagar *Salmonella* (CHROMagar, Paris, France) and Gram-negative broth (GNB; Dalynn Biologicals, Calgary, AB). If the CHROMagar was negative, the GNB was sub-cultured to a CHROMagar. Near the end of the study period, CHROMagar *Salmonella* was replaced with Xylose Lysine Deoxycholate agar (XLD; Dalynn) due to media availability in April 2022. Plates were incubated at 37°C for 18–24 hours and inspected for mauve (CHROMagar) or red colonies (XLD). *Salmonella* isolates were identified using MALDI-TOF and/or Vitek2 Gram Negative ID card. STEC stools were planted to CHROMagar STEC (CHROMagar, Paris, France) and GNB and incubated at 37°C for 18 to 24 hours. The presence of STEC was determined by a positive Shiga Toxin QUIK CHEK (TECHLAB Inc., Blacksburg, Virginia, USA) on mauve colonies from CHROMagar. If the CHROMagar was negative, QUIK CHEK was done from the GNB.

Initial diagnostic positive stools were reported to public health as per the Alberta Public Health Act. Specimens tested positive by BD MAX were frozen and retrospectively tested by lab-developed qPCR. In brief, frozen stools were thawed at 4°C, and a 20% stool suspension was prepared in phosphate saline buffer. A 250 µL aliquot of the stool suspension was used for DNA extraction using the MagaZorb DNA mini prep kit (Promega Corp., Wisconsin, USA) and KingFisher (Thermo Scientific, Vantaa, Finland) extraction instrument. The extracted DNA was analyzed by real-time PCR using primers and probes for *Salmonella* spp. (3), STEC (13), and *Campylobacter* spp. (14). Nucleic acid extraction and qPCR assay conditions were as in Thilakarathna et al. (15).

### Statistical analysis

Data were analyzed using GraphPad Prism Version 10.0. Survival curves were analyzed by the log-rank test. Study participants who did not have a documented negative BD MAX or culture result by the end of the study were censored. Statistical significance was set at *P* < 0.05.

## RESULTS

### Patient enrollment

Participants were enrolled between July 2019 and December 2022. During the study period, 1,526 positive patients were screened for eligibility (Fig. 1). Of 1,100 eligible patients, 83 were enrolled, and 37 (37/83, 44.6%) submitted at least one stool sample after the initial positive test. The mean number of stools submitted for all participants was 3.98 (range 1–5). Participant demographic data based on the organism is shown in Table 1.

**TABLE 1:** Demographic data and median shedding duration of participants based on stool test positivity by organism type

	Enteropathogens detected from the diagnostic stool specimen		
	*Campylobacter*	*Salmonella*	STEC
Number of patients	19	8	10
Mean age (range)	45.5 (18–72)	36.9 (24–50)	44.3 (23–67)
Gender (%female)	52.6% (10/19)	87.5% (7/8)	50% (5/10)
Mean duration of diarrhea in days (range)	7.3 (3–14)	7.5 (4–10)	7.3 (4–12)
Mean number of samples submitted per patient (range)	3.47 (1–5)	4.38 (1–5)	4.5 (2–5)
Number of patients hospitalized	0	0	1
Median duration of shedding by CIDT (days)	42	29	22
Median duration of shedding by culture (days)	29	29	19.5

### Campylobacter

**Fig 2:**
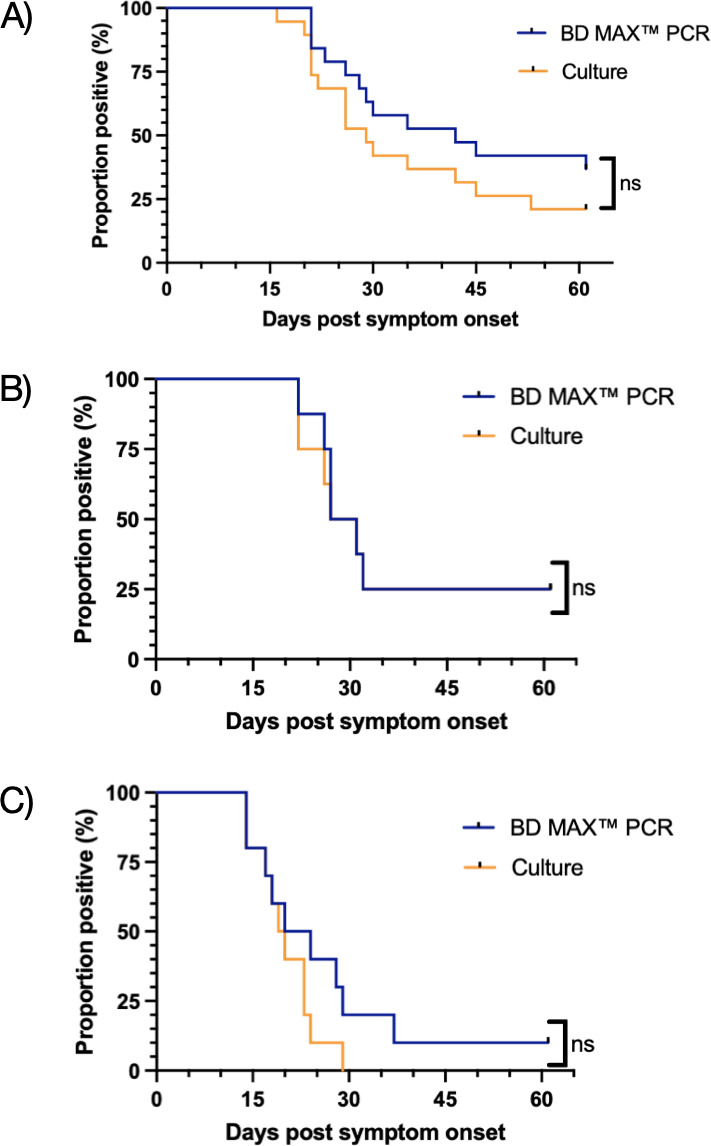
Clearance curves for stool. Colored lines represent proportions of samples that test positive by BD MAX and culture by organism (*Campylobacter* in panel A; *Salmonella* in panel B; STEC in panel C). Significance determined by log-rank test (ns= non-significant).

Nineteen *Campylobacter jejuni* or *C. coli-*positive individuals participated. Median duration of shedding was 42 and 29 days, for BD MAX and culture, respectively (Table 1; Fig. 2A). By the end of the study period, 68.4% (13/19) had a documented negative result. All patients with subsequent positive tests denied ongoing symptoms of diarrhea; however, the exact date of resolution is not known for all patients (Table S1). The agreement between culture and BD MAX varied from 75% to 100%, with 100% agreement in weeks 6–9 (Table 2), and the differences in the proportion of shedding detected by CIDT and culture over time were not statistically significant (Fig. 1).

**TABLE 2:** Agreement between CIDT and culture-tested specimens grouped by submission week

Organism	Week 2—excluded due to limited submissions	Week 3 (15–21 days)	Week 4 (22–28 days)	Week 5 (29–35 days)	Week 6 (36–42 days)	Weeks 7–9 (43–61 days)
*Campylobacter*	N/A^*a*^	13/17 = 76.4%	15/20 = 75%	11/13 = 84.6%	10/10 = 100%	5/5 = 100%
*Salmonella*	N/A	7/7 = 100%	7/8 = 87.5%	8/8 = 100%	8/8 = 100%	4/4 = 100%
STEC	N/A	6/7 = 85.7%	11/13 = 84.6%	8/8 = 100%	7/8 = 87.5%	6/7 = 85.7%

^*a*^ N/A=not analyzed.

Discordant results between culture and BD MAX were observed in 9/64 specimens (14.1%) representing seven unique patients. For discordant results, all cases were negative by culture and positive by BD MAX with cycle threshold (Ct) values greater than 29 (mean 32.2).

Of the 64 stools, 15 were missing qPCR data due to lab error. Agreement with confirmatory qPCR was 40/49 (81.6%), of which seven cases were BD MAX-negative and qPCR positive. The two remaining cases were BD MAX-positive and were undetectable by qPCR.

### Salmonella

Eight individuals positive for non-typhoidal *Salmonella* participated. Median duration of positivity for both BD MAX and culture was 29 days (Fig. 2B). By the end of the study period, 2/8 patients still tested positive by both methods at 43 and 44 days post-symptom onset for CIDT and culture, respectively. Differences in the proportion of shedding by BD MAX and culture over time were not statistically significant.

One discordant result (2.86%, *n* = 35) between BD MAX and culture was observed. The discordant specimen was culture-negative and BD MAX-positive, with a Ct value of 37.2. The qPCR Ct value for this sample was 33.9. The sample was the first sample submitted by this participant (22 days post-symptom onset), and symptoms had resolved 8 days post-onset (Table S1). Agreement with BD MAX and qPCR was 90.9% (30/33), with all specimens in disagreement being BD MAX-negative and qPCR positive. Out of the 35 stool samples, two specimens had missing qPCR results due to an error.

### Shiga toxin-producing *Escherichia coli*

Ten STEC-positive individuals participated. Median duration of shedding was 22 days by BD MAX and 19.5 days by culture (Fig. 2C). All 10 participants had negative stool culture before day 30 post-symptom onset. One participant remained BD MAX-positive (last sample submitted at 43 days) despite resolution of symptoms at day 12. The Ct value for this specimen was 34.2. Differences in the proportion of shedding by BD MAX and culture over time were not statistically significant.

Discordant results between BD MAX and culture were seen in 4 of 43 samples (9.1%). All discordant results were from a single participant with negative culture and positive BD MAX testing, with a mean Ct of 32.2 on the BD MAX. The participants’ symptoms were resolved by the time of collection of the samples with discordant results. Agreement between BD MAX and confirmatory qPCR was 90.5% (29/35). Eight specimens were not analyzed by confirmatory qPCR due to lab error. Of six discordant specimens, four specimens tested BD MAX-negative and qPCR-positive. The remaining two specimens tested positive by BD MAX with Ct values >30 and undetectable by qPCR.

### Correlation of CIDT and culture

**Fig 3:**
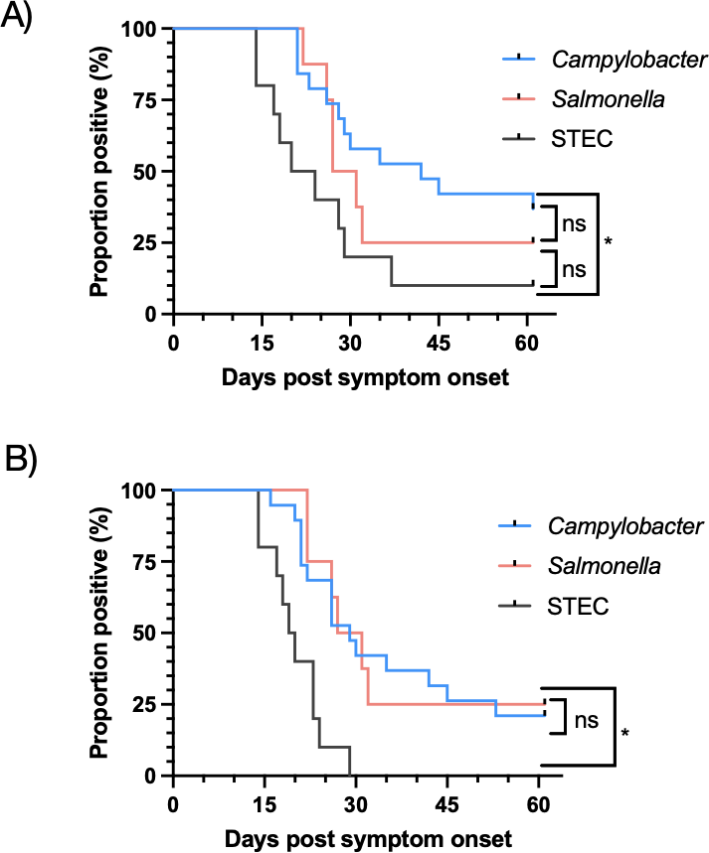
Proportion of shedding for all organisms by BD MAX (A) and culture (B). Clearance curves were analyzed by the log-rank test. ns, non-significant; *, statistically significant (panel A: *P* = 0.0141; panel B: *P* = 0.0003).

*Campylobacter* had the longest median shedding duration (42 days) when tested by CIDT. Survival curve analysis showed that the proportion of test positivity by BD MAX and culture was statistically significantly longer for *Campylobacter* than STEC, but not *Salmonella* (Fig. 3). STEC-infected participants demonstrated the fastest organism clearance by both tested methods, with a median of 19.5 days for culture and 22 days for BD MAX.

For all three organisms, the negative predictive value (NPV) of CIDT was 100% using culture results as the reference standard. Survival curve analysis demonstrated that the difference in positivity rates over time is not statistically significant between CIDT and culture for the three organisms (Fig. 3).

Percent agreement between CIDT (BD MAX) and culture was calculated for each tested organism categorized by weeks after symptom onset (Table 2). Week 2 (days 8–14) had only three specimen submissions between all participants and was not included in the analysis. Overall agreement for all specimens/organisms was 90.2% (129/143). Within weeks 3–7 post-symptom onset, the agreement between BD MAX and culture for *Campylobacter* was 75%–100%, for *Salmonella* was 87.5%–100%, and for STEC was 84.6%–100%.

All discordant results were CIDT-positive and culture-negative. Kappa scores could not be determined for all time points due to limitations of the data set. Agreement between BD MAX and culture was lowest during the first 3–4 weeks after symptom onset (75%–100%). During weeks 5–7, agreement was higher when most specimens were negative by both methods (range 80%–100%). Ct values for serial stool submissions over time per patient are shown in Table S1. Ct values generally increase over time for most stools tested.

### Lab-developed qPCR

Specimens that were BD MAX-negative but were qPCR-positive all had Ct values greater than 32 (*Campylobacter n* = 7, *Salmonella n* = 3, STEC *n* = 4). There were two cases of *Campylobacter* in which the BD MAX was positive and the qPCR was negative. The BD MAX-derived Ct values for these specimens were 30.6 and 30.1. Discrepant results occurred in weeks 3–7 (Table S1).

## DISCUSSION

In this study, we examined the kinetics of bacterial shedding in stool after an episode of gastroenteritis with culture and CIDT. It was expected that samples would remain CIDT-positive longer than culture due to the shedding of non-viable organisms; however, this was not demonstrated for *Campylobacter, Salmonella,* or STEC. Median duration of shedding by CIDT was longer than or equal to culture positivity for all three organisms, yet no statistically significant differences were observed. Though no differences in proportions of test positivity were found, the study was likely underpowered to detect significant differences in shedding between methods.

In our study, *Campylobacter* shed for the longest median duration. Data on shedding of *Campylobacter* species is mostly limited to veterinary studies (16). Our data suggest that *Campylobacter* may shed for a prolonged period in humans by both PCR-based and culture-based methods compared to other typical enteric pathogens. *Salmonella* was cleared by week 4 for six of eight participants. The other two participants were culture and CIDT-positive for *Salmonella* for the duration of the study. STEC cleared the most rapidly of our tested organisms, with 9/10 patients being CIDT-negative by 6 weeks post-initial diagnostic test. Recent studies have demonstrated STEC shedding for a median of 18 days (17) and 22–23 days (18), which is consistent with our results. In paediatric studies, STEC had a median culture clearance of 39 days, with 90% clearance by day 70 (19). Children have been shown to shed organisms longer than adults, emphasizing the importance of studying paediatric cohorts (19).

For all stool specimens submitted, the overall agreement between CIDT and culture was high at 90.2%. In all cases of disagreement, the discrepant specimens tested CIDT-positive and culture-negative. It is possible that the discrepancies, particularly for the more fastidious *Campylobacter,* may have been impacted by not using transport media; however, the discrepancy was not statistically significant. STEC and *Salmonella* recovery are unlikely to have been affected by not using transport media (20). In our study, a negative CIDT was 100% predictive for ruling out a positive culture. A recent study evaluated shedding after *Salmonella* infection, which demonstrated good overall agreement between PCR and culture, with high NPV for culture negativity in specimens that were PCR-negative (21). As negative culture is currently required for public health management (e.g., exclusion from sensitive situations or occupations), a definitive negative molecular test may replace the need for culture confirmation. Jääskeläinen et al. (18) demonstrated that two consecutive negative PCRs can replace culture for STEC and have subsequently updated Finnish national guidelines to reflect this. This demonstrates the important clinical utility of CIDT and could allow for quicker return to work in patients with resolved symptoms without the reliance on a negative culture, which may take several additional days. However, culture would still be necessary for the first specimen for typing and, when indicated, antibiotic susceptibility testing. Of note in our study, four participants had specimens that were culture and CIDT-negative with repeat submissions that were initially positive (*Campylobacter n* = 3, *Salmonella n* = 1) by both methods. Three of these participants had subsequent samples that were culture and CIDT-negative. One participant did not have additional specimens. Reasons for this fluctuation in positivity are unclear, but have been seen in other studies, specifically for STEC (18).

The lab-developed qPCR for each target organism was utilized as an external validation on each specimen received as part of the study. Overall, there was good agreement, but 16 specimens had discrepant results between BD MAX and qPCR. It is expected to have some discrepancies between PCRs, especially at the higher Ct values observed in discrepant results; importantly, all were culture-negative. Testing by qPCR required specimens to be shipped to a different laboratory, and the resultant asynchronous testing may explain the two discrepant results.

Limited submissions were received during the second week after diagnosis. The decision to collect stools starting in week 2 after infection was based on shedding literature (11, 22). A sizeable proportion of the cohort was culture and CIDT-negative by day 30; therefore, in future studies, it may be of benefit to include samples in the first 2 weeks of illness to better characterize early shedding of these pathogens.

The major limitation of our pilot study was the sample size. To better understand the nuances of shedding, a larger study size in a collaborative effort with other clinical laboratories, comparing other commercially available assays, would be of benefit. Another limitation was compliance with stool submission over the study period. The number and timing of submissions were highly variable between participants, making categorization and interpretation of data more challenging. Inconsistency in stool submissions may be improved upon by frequent reminders to participants or offering other methods of sample collection, such as rectal swabs. Further studies are also needed to characterize the impact of antimicrobials on shedding and CIDT vs culture performance. Despite these limitations, this was one of the first studies to demonstrate the kinetics of shedding by CIDT, which represents an important step toward a better understanding of molecular assays as they become more widely implemented in clinical laboratories and used by clinicians and public health practitioners.

## SUPPLEMENTAL MATERIAL

Supplemental material spectrum.00227-25-s0001.pdf The collection instructions sent to study participants.

Table S1 spectrum.00227-25-s0002.docx Individual culture and PCR results for each study participant and sample submitted.

ASM does not own the copyrights to Supplemental Material that may be linked to, or accessed through, an article. The authors have granted ASM a non-exclusive, world-wide license to publish the Supplemental Material files. Please contact the corresponding author directly for reuse.
