# Supplementary material for: Bacterial gastroenteritis in the world of culture-independent diagnostic testing: a study to evaluate the kinetics of bacterial shedding by culture and CIDT
Source: Microbiol Spectr. 2025 May 22;13(7):e00227-25. doi: 10.1128/spectrum.00227-25 (PMC12210883; doi:10.1128/spectrum.00227-25)
Supplement: Supplemental material — The collection instructions sent to study participants. [file spectrum.00227-25-s0001.pdf]

# Instructions for Stool Collection

**Drop off at any CLS Patient Service Centre or**
**Pick up available Monday to Friday between 8:00am to 3:30pm by calling 403-###-####.**
**Please reference Account #####.**
**Please be available to await pickup within 1.5 hours after calling.**

**STEP 1** –Collect the stool sample into any clean, dry disposable container or onto plastic wrap placed under the toilet seat, or waxed cardboard container.
***DO NOT let water touch the stool sample. ***

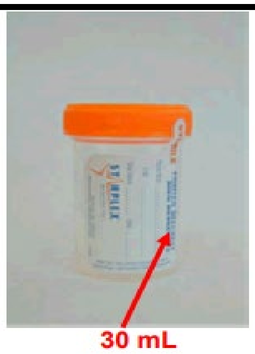


**STEP 2** - Fill the orange-capped empty container at least 1/3 full (30 mL). Do not fill more than half full.

**STEP 3** - WASH hands with soap and water. Write the date and time of collection one the label (Figure 2). If there is no Study ID label on the container, please write your name and date of birth on the label of the container.

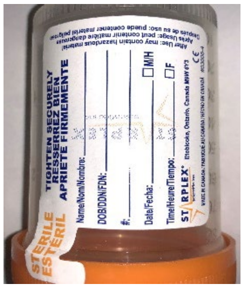

**STEP 4** - Put the Stool Sample Container in the plastic bag provided (labeled Stool Sample Container Bag) and seal (Figure 3). Wash hands with soap and water.
*Please note, the Stool Sample Container must be placed in a bag by itself, which can then be placed in the additional paper bag provided.

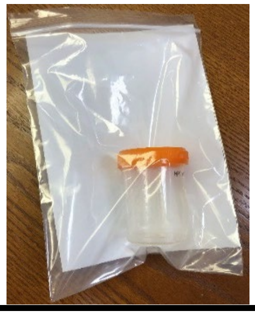

**STEP 5**- Fill out the "CLS Research Requisition". If it is the first time you have submitted stool also sign the consent form provided.

**STEP 6** - Place the plastic bag in the brown bag provided with the "CLS Research Requisition" and consent form. If you can, staple the bag shut.

**STEP 7 -** Drop the bag off at any Calgary Laboratory Services Patient Service Centre. If outside the Calgary area, drop it off at one of the locations provided in the package. No appointment is needed.
http://www.calgarylabservices.com/lab-patient/lab-locations/PSC-lab-locations.aspx

**OR** If you live within the Calgary area, call the courier (West Direct Courier) at 403-###-####, Account # ####, **as soon as possible** for sample pickup. Their daily hours are 8:00am to 3:30pm Monday to Friday, and they will come pick it up within 1.5 hours after calling.
If neither of these options work for you, West Direct is available outside of business hours but this will incur significant cost to the study. Please avoid doing this if possible.

**THANK YOU FOR YOUR PARTICIPATION**

If you have any questions, please contact
[Contact information removed for publication]
