# Supplementary material for: Bacterial gastroenteritis in the world of culture-independent diagnostic testing: a study to evaluate the kinetics of bacterial shedding by culture and CIDT
Source: Microbiol Spectr. 2025 May 22;13(7):e00227-25. doi: 10.1128/spectrum.00227-25 (PMC12210883; doi:10.1128/spectrum.00227-25)
Supplement: Table S1 — Individual culture and PCR results for each study participant and sample submitted. [file spectrum.00227-25-s0002.docx]

Supplementary Table 1: Individual culture and PCR results for each study participant and sample submitted

| **Organism** | **Study ID** | **Sample #** | **Sample collection after symptom onset (days)** | **Culture Result** | **BD MAX Result** | **BD MAX CT value** | **qPCR CT (mean of triplicate)**  **n/a=not available** |
| --- | --- | --- | --- | --- | --- | --- | --- |
| ***Campylobacter*** | **2** | 1 | 22 | + | + | 20.8 | 22.16 |
|  |  |  |  |  |  |  |  |
|  | **3** | 1 | 16 | - | + | 35.0 | 27.97 |
|  |  | 2 | 23 | - | - | - | - |
|  |  |  |  |  |  |  |  |
|  | **8** | 1 | 16 | + | + | 28.2 | 26.66 |
|  |  | 2 | 22 | + | + | 30.0 | 31.02 |
|  |  |  |  |  |  |  |  |
|  | **15** | 1 | 19 | + | + | 22.3 | 23.03 |
|  |  | 2 | 21 | + | + | 20.4 | 23.96 |
|  |  | 3 | 28 | + | + | 22.7 | 26.31 |
|  |  | 4 | 38 | + | + | 22.1 | n/a |
|  |  | 5 | 42 | + | + | 27.3 | n/a |
|  |  |  |  |  |  |  |  |
|  | **16** | 1 | 22 | - | + | 29.2 | 32.78 |
|  |  | 2 | 28 | - | + | 31.9 | 30.28 |
|  |  | 3 | 35 | - | + | 31.3 | 30.17 |
|  |  |  |  |  |  |  |  |
|  | **18** | 1 | 21 | + | + | 31.9 | 26.89 |
|  |  | 2 | 26 | - | - | - | - |
|  |  | 3 | 27 | - | - | - | - |
|  |  | 4 | 34 | - | - | - | - |
|  |  | 5 | 45 | - | - | - | - |
|  |  |  |  |  |  |  |  |
|  | **19** | 1 | 18 | - | - | - | - |
|  |  | 2 | 25 | + | + | 27.4 | 30.07 |
|  |  | 3 | 28 | + | + | 28.3 | 23.03 |
|  |  | 4 | 39 | - | - | - | - |
|  |  |  |  |  |  |  |  |
|  | **24** | 1 | 15 | + | + | 25.1 | 25.67 |
|  |  | 2 | 21 | + | + | 25.5 | 27.88 |
|  |  | 3 | 28 | + | + | 25.6 | 27.07 |
|  |  | 4 | 35 | + | + | 27.2 | n/a |
|  |  |  |  |  |  |  |  |
|  | **27** | 1 | 21 | - | - | - | - |
|  |  | 2 | 28 | - | - | - | 38.27 |
|  |  | 3 | 35 | - | - | - | n/a |
|  |  | 4 | 42 | - | - | - | n/a |
|  |  |  |  |  |  |  |  |
|  | **31** | 1 | 21 | - | - | - | - |
|  |  | 2 | 28 | - | - | - | 37.71 |
|  |  | 3 | 35 | - | - | - | - |
|  |  | 4 | 41 | - | - | - | n/a |
|  |  |  |  |  |  |  |  |
|  | **40** | 1 | 20 | - | + | 32.8 | 35.47 |
|  |  | 2 | 23 | + | + | 25.4 | 28.15 |
|  |  | 3 | 28 | + | + | 30.6 | - |
|  |  | 4 | 35 | - | - | - | n/a |
|  |  | 5 | 42 | - | - | - | n/a |
|  |  |  |  |  |  |  |  |
|  | **43** | 1 | 20 | - | + | 34.6 | 36.11 |
|  |  | 2 | 21 | - | - | - | 28.15 |
|  |  | 3 | 28 | + | + | 30.1 | - |
|  |  | 4 | 36 | - | - | - | n/a |
|  |  | 5 | 42 | - | - | - | n/a |
|  |  |  |  |  |  |  |  |
|  | **48** | 1 | 14 | + | + | 20.9 | 33.72 |
|  |  | 2 | 21 | - | - | - | 34.49 |
|  |  | 3 | 35 | - | - | - | n/a |
|  |  | 4 | 43 | - | - | - | n/a |
|  |  |  |  |  |  |  |  |
|  | **51** | 1 | 26 | - | + | 29.3 | n/a |
|  |  |  |  |  |  |  |  |
|  | **59** | 1 | 16 | - | - | - | 32.56 |
|  |  | 2 | 21 | - | - | - | - |
|  |  | 4 | 35 | - | - | - | n/a |
|  |  | 5 | 42 | - | - | - | n/a |
|  |  |  |  |  |  |  |  |
|  | **64** | 1 | 29 | - | - | - | - |
|  |  | 2 | 33 | - | - | - | 38.39 |
|  |  |  |  |  |  |  |  |
|  | **70** | 1 | 25 | + | + | 31.2 | 33.38 |
|  |  | 2 | 26 | - | + | 34.2 | 33.64 |
|  |  | 3 | 30 | - | - | - | - |
|  |  | 4 | 45 | - | - | - | - |
|  |  |  |  |  |  |  |  |
|  | **73** | 1 | 30 | - | + | 31.3 | 33.73 |
|  |  | 2 | 61 | - | - | - | 36.58 |
|  |  |  |  |  |  |  |  |
|  | **76** | 1 | 35 | + | + | 31.6 | 34.69 |
|  |  | 2 | 53 | + | + | 31.5 | 33.54 |
|  |  |  |  |  |  |  |  |
| ***Salmonella*** | **7** | 1 | 18 | - | - | - | 33.67 |
|  |  | 2 | 21 | + | + | 33.3 | 28.67 |
|  |  | 3 | 27 | - | - | - | - |
|  |  | 4 | 35 | - | - | - | - |
|  |  | 5 | 45 | - | - | - | - |
|  |  |  |  |  |  |  |  |
|  | **14** | 1 | 17 | + | + | 22.2 | 18.19 |
|  |  | 2 | 21 | + | + | 24.2 | 17.71 |
|  |  | 3 | 31 | + | + | 24.1 | 20.07 |
|  |  | 4 | 36 | + | + | 20.2 | 29.37 |
|  |  | 5 | 44 | + | + | 24.7 | 21.14 |
|  |  |  |  |  |  |  |  |
|  | **25** | 1 | 20 | + | + | 23.6 | 20.20 |
|  |  | 2 | 21 | + | + | 22.3 | 20.68 |
|  |  | 3 | 28 | + | + | 25.7 | 18.21 |
|  |  | 4 | 35 | + | + | 20.7 | 22.70 |
|  |  | 5 | 43 | + | + | 23.4 | n/a |
|  |  |  |  |  |  |  |  |
|  | **36** | 1 | 21 | - | - | - | - |
|  |  | 2 | 28 | - | - | - | - |
|  |  | 3 | 34 | - | - | - | - |
|  |  | 4 | 41 | - | - | - | - |
|  |  | 5 | 42 | - | - | - | n/a |
|  |  |  |  |  |  |  |  |
|  | **42** | 1 | 26 | - | - | - | 33.56 |
|  |  | 2 | 28 | - | - | - | - |
|  |  | 3 | 30 | - | - | - | - |
|  |  | 4 | 35 | - | - | - | - |
|  |  | 5 | 42 | - | - | - | - |
|  |  |  |  |  |  |  |  |
|  | **44** | 1 | 22 | - | - | - | - |
|  |  |  |  |  |  |  |  |
|  | **61** | 1 | 22 | - | + | 37.2 | 33.89 |
|  |  | 2 | 27 | - | - | - | - |
|  |  | 3 | 31 | - | - | - | - |
|  |  | 4 | 37 | - | - | - | - |
|  |  | 5 | 42 | - | - | - | - |
|  |  |  |  |  |  |  |  |
|  | **69** | 1 | 32 | - | - | - | - |
|  |  | 2 | 39 | - | - | - | - |
|  |  | 3 | 41 | - | - | - | - |
|  |  | 4 | 66 | - | - | - | 34.60 |
|  |  |  |  |  |  |  |  |
| **STEC** | **12** | 1 | 14 | - | - | - | 34.05 |
|  |  | 2 | 21 | - | - | - | - |
|  |  | 3 | 28 | - | - | - | - |
|  |  | 4 | 34 | - | - | - | - |
|  |  | 5 | 41 | - | - | - | - |
|  |  |  |  |  |  |  |  |
|  | **17** | 1 | 18 | - | - | - | 33.247 |
|  |  | 2 | 28 | - | - | - | - |
|  |  | 3 | 34 | - | - | - | - |
|  |  | 4 | 41 | - | - | - | - |
|  |  | 5 | 49 | - | - | - | - |
|  |  |  |  |  |  |  |  |
|  | **21** | 1 | 14 | - | - | - | - |
|  |  | 2 | 21 | - | - | - | - |
|  |  | 3 | 28 | - | - | - | - |
|  |  | 4 | 35 | - | - | - | - |
|  |  | 5 | 42 | - | - | - | - |
|  |  |  |  |  |  |  |  |
|  | **23** | 1 | 28 | - | - | - | n/a |
|  |  | 2 | 33 | - | - | - | n/a |
|  |  | 3 | 38 | - | - | - | n/a |
|  |  |  |  |  |  |  |  |
|  | **26** | 1 | 20 | - | - | - | - |
|  |  | 2 | 27 | - | - | - | 35.838 |
|  |  | 3 | 35 | - | - | - | n/a |
|  |  | 4 | 41 | - | - | - | n/a |
|  |  | 5 | 48 | - | - | - | n/a |
|  |  |  |  |  |  |  |  |
|  | **45** | 1 | 19 | - | - | - | 33.462 |
|  |  | 2 | 24 | - | - | - | - |
|  |  | 3 | 28 | - | - | - | - |
|  |  | 4 | 35 | - | - | - | - |
|  |  | 5 | 44 | - | - | - | - |
|  |  |  |  |  |  |  |  |
|  | **47** | 1 | 23 | - | - | - | - |
|  |  | 2 | 25 | - | - | - | - |
|  |  | 3 | 30 | - | - | - | - |
|  |  | 4 | 37 | - | - | - | - |
|  |  | 5 | 44 | - | - | - | - |
|  |  |  |  |  |  |  |  |
|  | **54** | 1 | 23 | - | - | - | - |
|  |  | 2 | 26 | - | + | 30.6 | 38.776 |
|  |  | 3 | 28 | - | + | 30.5 | - |
|  |  | 4 | 36 | - | + | 33.6 | - |
|  |  | 5 | 43 | - | + | 34.2 | n/a |
|  |  |  |  |  |  |  |  |
|  | **74** | 1 | 29 | - | - | - | - |
|  |  | 2 | 42 | - | - | - | - |
|  |  | 3 | 48 | - | - | - | - |
|  |  | 4 | 56 | - | - | - | - |
|  |  |  |  |  |  |  |  |
|  | **83** | 1 | 24 | - | - | - | n/a |
